# Infrastructure features outperform environmental variables explaining rabbit abundance around motorways

**DOI:** 10.1002/ece3.3709

**Published:** 2017-12-12

**Authors:** Aimara Planillo, Juan E. Malo

**Affiliations:** ^1^ Terrestrial Ecology Group (TEG) Department of Ecology Universidad Autónoma de Madrid Madrid Spain

**Keywords:** altered landscapes, anthropogenic disturbance, environmental impact assessment, mitigation, *Oryctolagus cuniculus*, road ecology

## Abstract

Human disturbance is widespread across landscapes in the form of roads that alter wildlife populations. Knowing which road features are responsible for the species response and their relevance in comparison with environmental variables will provide useful information for effective conservation measures. We sampled relative abundance of European rabbits, a very widespread species, in motorway verges at regional scale, in an area with large variability in environmental and infrastructure conditions. Environmental variables included vegetation structure, plant productivity, distance to water sources, and altitude. Infrastructure characteristics were the type of vegetation in verges, verge width, traffic volume, and the presence of embankments. We performed a variance partitioning analysis to determine the relative importance of two sets of variables on rabbit abundance. Additionally, we identified the most important variables and their effects model averaging after model selection by AICc on hypothesis‐based models. As a group, infrastructure features explained four times more variability in rabbit abundance than environmental variables, being the effects of the former critical in motorway stretches located in altered landscapes with no available habitat for rabbits, such as agricultural fields. Model selection and Akaike weights showed that verge width and traffic volume are the most important variables explaining rabbit abundance index, with positive and negative effects, respectively. In the light of these results, the response of species to the infrastructure can be modulated through the modification of motorway features, being some of them manageable in the design phase. The identification of such features leads to suggestions for improvement through low‐cost corrective measures and conservation plans. As a general indication, keeping motorway verges less than 10 m wide will prevent high densities of rabbits and avoid the unwanted effects that rabbit populations can generate in some areas.

## 1 INTRODUCTION

As human population grows, human pressure on nature increases and reaches more areas. Terrestrial ecosystems are increasingly altered by anthropogenic activities, affecting to some degree more than 80% of the land surface (Foley et al., 2005; Sanderson et al., 2002). Human activities modify the conditions under which wildlife populations develop (Clavel, Julliard, & Devictor, 2011; Hobbs et al., 2006), and therefore, species response to disturbance is of great interest from a conservation point of view.

One way to minimize ecosystems' alterations is the identification of key features that are responsible for wildlife populations' response to disturbance, which will allow the development of effective conservation actions as well as optimize the cost of mitigation measures (van der Grift et al., 2013; Malo, Suarez, & Diez, 2004; Ward, Dendy, & Cowan, 2015). Wildlife populations in road verges respond to road features and habitat characteristics (Clevenger, Chruszczc, & Gunson, 2003; Farmer & Brooks, 2012; Santos, Lourenço, Mira, & Beja, 2013). However, for a better understanding of the species response, we need to disentangle the effects of different factors and assess which of those factors have the strongest impacts. Furthermore, in the case of anthropogenic disturbance, it is interesting to know to what extend and which modifications introduced by human activities are responsible for observed changes in wild species. By identifying such features, we can anticipate which actions are expected to affect populations of target species and to give specific recommendations to managers and policy makers to design conservation plans.

An incomparable case of human disturbance is road infrastructure, which have a huge impact on wildlife populations (Benítez‐López, Alkemade, & Verweij, 2010). Roads are widespread around the world, affecting many different ecosystems, and their effects expand over large areas around them (Forman et al., 2003; van der Ree, Smith, & Grilo, 2015; Torres, Jaeger, & Alonso, 2016). For example, in the United States, at least the 22% of the land area is less than 100 m from a road and 80% is within 1 km (Forman, 2000; Riitters & Wickham, 2003). The road density is even higher in Europe, and prospects are for its increase worldwide (Dulac, 2013).

Among many others effects, an interesting aspect of roads is that they provide new habitat and corridors that are used by small mammals (Galantinho et al., 2017; de Redon et al., 2015; Ruiz‐Capillas, Mata, & Malo, 2013). However, this effect may not be desirable in every situation, as it can act as corridor for invasive species (Downes, Handasyde, & Elgar, 1997; Trombulak & Frissell, 2000), and in some situations, dense populations of prey near roads could attract predators and increase their mortality risk (Barrientos & Bolonio, 2009; Grilo, Reto, Filipe, Ascensão, & Revilla, 2014; Planillo, Mata, Manica, & Malo, in press).

**Figure 1:**
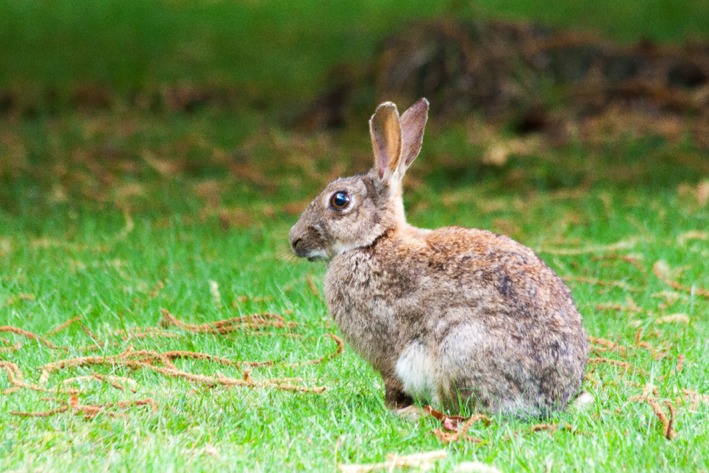
European rabbit (*Oryctolagus cuniculus*)

The European rabbit *Oryctolagus cuniculus* (Figure 1) is a key prey species in Mediterranean ecosystems and classified as “vulnerable” in Spain (Delibes‐Mateos, Delibes, Ferreras, & Villafuerte, 2008; Gálvez‐Bravo, López‐Pintor, Rebollo, & Gómez‐Sal, 2011; Villafuerte & Delibes‐Mateos, 2007), and this species has the ability to use roadside verges as habitats as well as corridors to spread through the landscape (Bautista et al., 2004; Planillo & Malo, 2013). Within its native range, rabbits are the preferred prey for most of the Iberian vertebrate predators and therefore, a species of conservation concern (Delibes‐Mateos et al., 2008; Virgós, Cabezas‐Díaz, & Lozano, 2007). Meanwhile, in other areas of the world, rabbits are invasive species that spread quickly in new environments, causing severe damage to the ecosystems they invade (Lees & Bell, 2008). These characteristics make the European rabbit the optimal species for this study due to their implications in human‐altered landscapes: conservation problems in native range, plague, or invasive species in several areas of the world, as well as responsible for damages to infrastructures due to their digging activity.

In this study, we analyze variability in rabbit abundance in the human‐altered habitat of motorway verges within its native range, with three main objectives: (1) assess the relative importance of factors related to the infrastructure (man‐related) in relation to those related to the natural environment; (2) determine which specific features are correlated with rabbit abundance in motorway verges; (3) propose measures to manage rabbit abundance in verges.

## 2 MATERIALS AND METHODS

### 2.1 Study area

**Figure 2:**
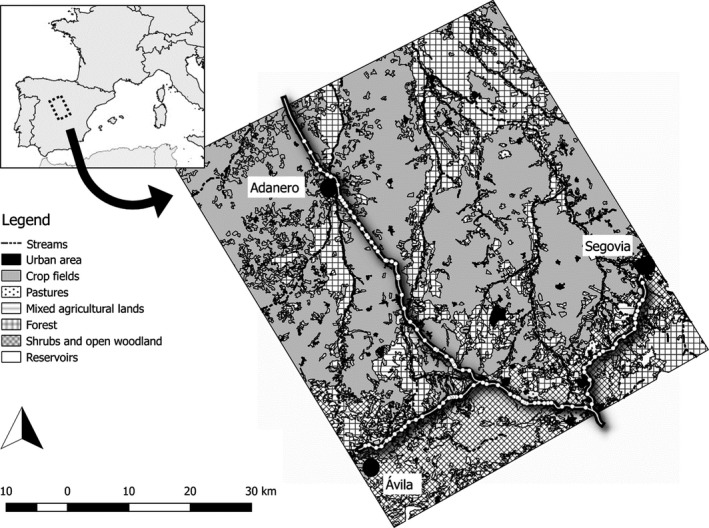
Study area in Central Spain. Motorways studied are highlighted in the landscape. White dots represent the location of sampling transects. Main vegetation types in the landscape are shown (see legend for details)

We conducted our study in Central Spain, provinces of Ávila and Segovia (40°46′N 4°25′W), in an area of around 3,000 km^2^ (55 × 55 km) which includes a wide range of environmental conditions (Figure 2 and Table S1). This region has a continental Mediterranean climate, with cold winters and an average rainfall of 450 mm a year, mainly in autumn and spring. The study area includes a gradient of environmental conditions, from open oak forest managed for cattle (“dehesas”) in the southern part to intensive nonirrigated field crops in the north, with a transition of mixed landscapes composed of small field crops and patches of natural vegetation. Three motorways are found in the study area, all of them fenced. Their main characteristics are summarized in Table 1.

**Table 1:** Description of the motorways in the study area, including the predominant vegetation (main vegetation) and the characteristics of the perimeter fence around each one

Motorway	Lengh (km)	Route	Lanes	Traffic^a^	Main Vegetation	Perimeter fence
AP‐6	27	Villacastín–Adanero	4	18,866	Small patches of natural vegetation and field crops	2 m tall progressive fence^b^
AP‐6	20	San Rafael–Villacastín	4	29,155	Natural vegetation and grasslands	2 m tall progressive fence^b^
A‐6	30	Adanero–Ataquines	4	31,284	Intensive field crops	1.5 m tall progressive fence^b^
AP‐61	28	San Rafael–Segovia	4–6	6,294	Oak forest and dehesas	2 m tall progressive fence^b^ with reinforcement^c^ and pinned^d^ to the ground
AP‐51	25	Villacastín–Ávila	4–6	8,450	Dehesas and field crops	2 m tall progressive fence^b^, with reinforcement^c^ and pinned^d^ to the ground

a Average daily traffic (ADT, vehicles per day) in 2009.

b Progressive fence composed of wired mesh of 15 × 5 to 15 × 20 cm.

c Reinforcement of 5 × 5 cm mesh in the lower part, up to 0.5 m.

d Pinned: The fence is buried underground.

### 2.2 Survey design and data collection

In spring 2010, we generated 100 random points—20 for each motorway section in Table 1—and we estimated an index of relative rabbit abundance. We did all the field surveys for rabbit abundance in the shortest time frame possible, from mid‐March to mid‐April, in order to avoid differences in rabbit abundance among motorways due to reproduction or mortality. High local mortality was ruled out as we did not detect any disease outbreak and mortality rates by roadkill were low in all the study area during our survey (unpublished results). Random points were separated by a minimum distance of 300 m so that a rabbit territory did not overlap two sampling points (home range 1 ha; Lombardi, Fernandez, & Moreno, 2007). Usually, rabbits live in breeding groups that can be formed by several males and females sharing a territory, which they defend from other groups (Cowan 1987), thus home range of an individual is expected to coincide with the territory size. From each point, we walked a 2 × 50 m transect and estimated a rabbit abundance index by counting pellets groups, following the method described in Fa, Sharples, and Bell (1999). This method is based on counting groups of pellets, divided into five size classes: class 1 = 1–2 pellets, class 2 = 3–15 pellets, class 3 = 16–50 pellets, class 4 = 51–150 pellets, and class 5 = >150 pellets. The abundance index is defined as the pellet density, estimated by summing the results of multiplying the number of groups of each size class by the mean number of pellets in that size class (Rabbit abundance index = c_1 _× 1.5 + c_2_ × 9 + c_3_ × 33 + c_4_ × 100 + c_5_ × 150, where c_*i*_ = number of groups of class *i*). The higher the value of the abundance index, a higher density of rabbits is assumed in an area. In an study comparing different rabbit abundance indices carried out in Spain, values of pellet counts of around 100 pellets m^−2^ were related to abundances of two rabbits ha^−1^ (Fernandez‐de‐Simon et al., 2011).

We opted for this method because the landscape near motorways in our study area was divided into small private fields, making difficult the use of longer transects. In addition, the presence of cattle prevented us from using standing plots, as cattle frequently remove marks from one visit to the next one.

**Figure 3:**
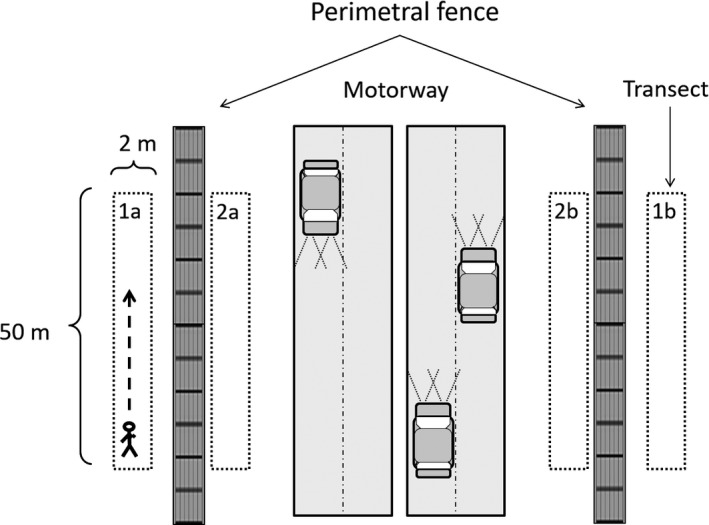
Survey design for estimating the rabbit abundance index. Detail of each motorway stretch with four parallel transects, two outside the perimeter fence (transects 1a and 1b) and two inside (transects 2a and 2b)

To properly characterize the rabbit abundance in each motorway stretch, we designed a system of four transects at each sampling point. Two transects on each side of the motorway, one outside and one inside the perimeter fence (Figure 3). Rabbit relative abundance index was estimated for each transect.

Rabbit abundance in motorway verges may respond to characteristics of either the landscape or the motorway, and thus, we divided explanatory variables into two groups: variables related to the environment (environmental variables hereafter), and variables related to the infrastructure (infrastructure variables hereafter). Environmental variables are descriptive of the landscape and may be seen as the underlying natural variables that shape the distribution and abundance of the species, usually out of management capabilities of road managers. On the contrary, infrastructure variables depend on the design of the motorways and some of them can be modified in response to management objectives.

#### 2.2.1 Environmental variables

We measured the main habitat characteristics in the environment surrounding the motorway outside the fenced area that could affect rabbit abundance (Table S1): altitude (“altitude”), cover of each vegetation type (simplified by PCA, see Data Analysis), vegetation productivity measured by NDVI (“ndvi”), and distance to closest river (“dist.water”). Initially, a measure of soil hardness was also taken, but the soft sandy soils of the area are not a limitation for rabbit burrowing (Rogers & Myers, 1979). As the values of soil hardness did not show any variation across the area, this resulted in a variable that is statistically nonrelevant, and thus, we opted for removing it from the analysis to avoid convergence problems and biased results. We used altitude as a proxy for climate, as our study area presents a Mediterranean climate and altitude is negatively related to temperature and positively related to rainfall, both factors affecting rabbit populations (Calvete, Estrada, Angulo, & Cabezas‐Ruiz, 2004; Palomares, 2003). We characterized the vegetation in 50‐m plots around each transect by two variables: the cover percentage of vegetation types (herbaceous, shrubs, trees, crops, and unproductive, for areas without vegetation cover) and the productivity of the vegetation measured by the Normalized Difference Vegetation Index (NDVI). Both variables were estimated by aerial images in a public repository of Castilla y León Government that are taken during summer (Junta de Castilla y León 2011). Finally, rivers are used as corridors by many species (Matos, Santos, Palomares, & Santos‐Reis, 2009; Sabino‐Marques & Mira, 2011; Virgós, 2001), thus wildlife species will have easier access to areas near them.

#### 2.2.2 Infrastructure variables

We estimated five variables describing the motorway and the conditions in the verges that may affect rabbit abundance (Table S1): traffic volume (“adt”), the width of the verges (“verge.width”), the presence of embankment (“embank”), and the cover of different types of vegetation within the fenced area: herbaceous, shrubs, trees, and unproductive. The traffic volume has been related to disturbance and mortality (Benítez‐López et al., 2010; Clarke, White, & Harris, 1998; Forman et al., 2003), and it was provided as average daily traffic (ADT) by the company responsible for the motorways (Iberpistas S.A.). We estimated the cover percentage of the vegetation classes in the whole verge parallel to each transect using aerial photographs. The width of the verges is a measure of the amount of habitat provided by the infrastructure and was defined as the distance from the asphalt surface to the perimeter fence. We took the distance in meters in the center of each sampling transect. Finally, we included the presence of embankment (“embank”) if the road was over the ground level at least in one side of the motorway. In our case, embankments covered the full length of the transect when present, thus we used the presence/absence of embankment instead of its length in each transect. Embankments may be important resources for rabbits because they provide potential ground for warren building in an elevated area that prevents flooding during rain periods.

### 2.3 Data analysis

From the 100 transects for surveying rabbit abundance, one transect was removed from the analyses because verges seemed under maintenance work, which could affect rabbit numbers and make results unreliable.

Prior to the analyses, we checked that the data obtained from transects outside and inside the perimeter fence (Figure 3) were comparable. This was carried out mostly by two reasons: First, working inside the fence is dangerous and forbidden in many areas, and second, outside transects are easier to access and replicate if desired. We compared data from both types of transects by a Spearman correlation and obtained a value of rho = 0.91, *p *<* *.001 (Figure S1), with fewer pellets found inside the fenced area (*n* = 99). These results indicate that information from transects inside and outside the perimeter fence was similar and probably coming from the same rabbit groups, thus we decided to use only values of outside transects for further analyses.

To characterize each motorway stretch, we computed the mean value of all variables measured in both sides. The cover percentages of vegetation types in the landscape outside the fence were simplified by a PCA to summarize the complex landscape and reduce the number of variables in the analyses. We used the first two axes as a summary of vegetation (Figure S2): Axis 1 distinguished between the abundance of crops (positive values) and cover of herbs and shrubs (negative values), and Axis 2 represented a gradient between unproductive areas (positive values) and high tree cover (negative values). The cover percentages of vegetation types inside the fenced area were highly correlated (percentage of herb cover was negative correlated with percentages of shrub and tree covers). As the vegetation structure inside the fence was quite simple, we opted to use only one variable in the analysis (cover of herbaceous vegetation, “cover.herb”), instead of using the complex axis of a PCA to help with interpretation of the variables.

We tested the correlation between explanatory variables and no correlation higher than 0.7 was found, thus all variables were included in the analyses. The linearity of the relationship between explanatory variables and rabbit abundance was inspected by univariate plots, and explanatory variables were transformed when necessary. Distances were log‐transformed, and proportions were transformed by arcsin (√x). Also, traffic volume suggested a quadratic relationship, and thus, it was introduced in the model as a quadratic effect. The response variable was transformed by square‐root transformation to meet the normality criterion. All explanatory variables were standardized to make their coefficients comparable (Quinn & Keough, 2002).

We analyzed the explanatory power of each group of variables, using variance partitioning analysis (Borcard, Legendre, & Drapeau, 1992; Ferrer‐Castán & Vetaas, 2005). For this analysis, we used three groups of variables: environmental, infrastructure, and spatial variables to account for the spatial distribution of data. For the environmental and infrastructure groups, we included only those variables that were found to have an effect on rabbit abundance. Spatial information was first tested on the response variable using a cubic regression and simplified using backward selection. Only the relevant spatial variables selected after the backward selection were used in the variance partition analysis (Borcard et al., 1992).

Prior to model building, we tested for spatial autocorrelation in rabbit abundance by Moran correlogram and found positive correlation. To account for this in the models, we used generalized least squares (GLS) regression models with spherical structure for spatial autocorrelation based on the semivariogram values (Beale, Lennon, Yearsley, Brewer, & Elston, 2010; Dormann et al., 2007).

We constructed GLS models according to hypothesis explaining rabbit abundance and compared them using Akaike information criterion corrected for small sample size AICc (Burnham & Anderson, 2002; Burnham, Anderson, & Huyvaert, 2011). The relative importance of each variable was measured as the sum of Akaike weights (*w*_*i*_) of the models containing that variable and the relative effect size of each variable is represented by the coefficient of the averaged model (Burnham & Anderson, 2002). Those variables for which the 85% confidence interval of the coefficient included the 0 were assumed to have no effect (Arnold, 2010).

The model set of hypothesis explaining rabbit abundance contained 13 models, belonging to the following hypothesis: (1) environmental characteristics outside the motorway fence; (2) motorway characteristics inside the area delimited by the perimetral fence; (3) vegetation structure (all vegetation, vegetation outside the fence, vegetation inside the fence), (4) refuge (verge width, the presence of embankment); (5) disturbance (traffic); (6) general climatic conditions; (7) distance to the nearest river; and (8) full model and null model for comparison (the full list of models can be seen in Table 2). In order to obtain a balanced model set, each explaining variable is contained in a similar number of models.

**Table 2:** Model selection table. Models based on hypothesis are ranked based on their AICc values (*k*: number of parameters; ΔAICc: difference between a given model and the model with the lowest AICc value; *w*_*i*_: AICc weight)

Model	Variables	*k*	AICc	ΔAICc	*w*_i_
Disturbance and refuge	adt+adt^2^+verge.width	4	207.8	0	0.587
Infrastructure	adt+adt^2^+cover.herb+embank+verge.width	6	208.7	0.87	0.38
Full model	adt+adt^2^+axis1+axis2+ndvi+ altitude+dist.water+ cover.herb+embank+verge.width	11	215	7.17	0.016
Refuge 1	verge.width	2	215.7	7.89	0.011
Vegetation (inside fence)	cover.herb	2	218.2	10.39	0.003
Vegetation (all)	cover.herb+axis1+axis2+ndvi	5	220.5	12.7	0.001
Disturbance	adt+adt^2^	3	220.8	13	0.001
Null model	—	1	223.6	15.81	<0.001
Clima	altitude	2	225	17.2	<0.001
Vegetation (outside fence)	axis1+axis2+ndvi	4	225.2	17.41	<0.001
Distance to water	dist.water	2	225.6	17.8	<0.001
Refuge 2	embank	2	225.7	17.84	<0.001
Environment	axis1+axis2+ndvi+altitude+dist.water	6	227.1	19.28	<0.001

All analyses were performed in R 3.3.3 (R Core Team 2017), using libraries vegan for variance partitioning (Oksanen et al., 2017), nlme for GLS models (Pinheiro, Bates, DebRoy, Sarkar, & R Core Team, 2017), and mumin for model averaging (Barton, 2017).

## 3 RESULTS

**Figure 4:**
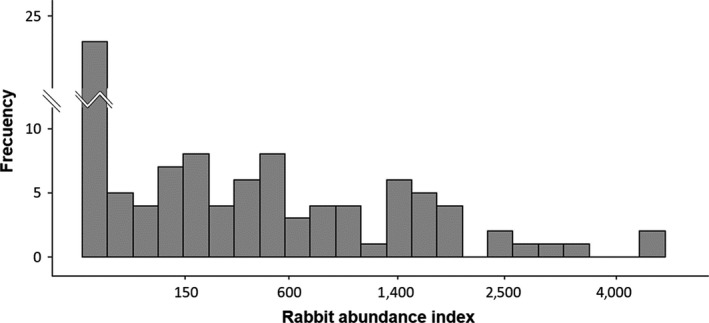
Frequencies of rabbit abundance index found in the 99 motorway stretches surveyed. Note that the vertical axis is discontinued

Our results from transects revealed abundance index values ranging from 0 to 4,662 (Figure 4), which mean pellets densities up to 40 pellets m^−2^. Obtained values covered a wide spectrum of abundances, from total absence to approx. 1–2 rabbits ha^−1^.

**Figure 5:**
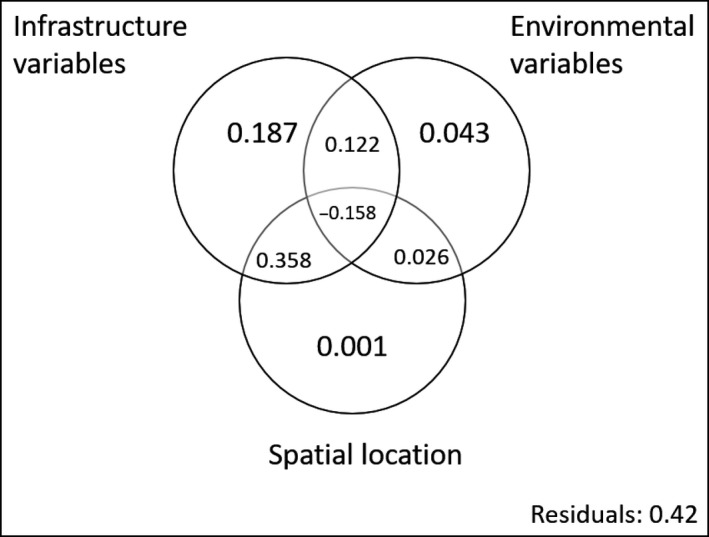
Variance partitioning of rabbit abundance in motorway verges among environmental‐, spatial‐, and infrastructure‐related variables. Large numbers in circles are proportions explained by each group of variables independently while shared portions among them are shown in the overlap regions

In the variance partitioning analysis (Figure 5), the environmental variables were those with less explanatory power, accounting only for 3% of the total variance, when taking into account the shared fractions. In contrast, the group of infrastructure variables explained 50.9% of the variance in rabbit abundance. The spatial location was the second most informative group, explaining 22.7% of variance, although most of it was shared with the infrastructure variables, as the infrastructure is inherently spatially continuous. Looking at the pure effect of the groups—without the shared fraction—infrastructure variables explained four times the variance explained by environmental ones (18.7% and 4.3%, respectively).

The model selection of the GLS models for rabbit abundance is summarized in Table 2. Following AICc selection, models that better explained rabbit abundance were the “disturbance and refuge” and the infrastructure characteristics models with small difference in their AICc. Less supported models were the full model and the refuge (verge width) model, with a difference of more than 7 AICc points. The remaining models presented a difference of more than 10 AICc points with the best candidate models, thus they should not be considered for interpretation.

Regarding the relative importance of the variables, variables of the infrastructure presented higher Akaike weights and higher coefficients, pointing to stronger effects (Table 3). Among them, the most important ones were the width of the verge and the traffic volume, with opposite effects, positive for wider verges, and negative for higher traffic volumes, as shown by the negative coefficient of the quadratic variable for traffic. Regarding the other two infrastructure variables, the cover of herbs in the verge presented a positive relation with rabbit abundance index, although being less important than traffic and verge width. And the presence of embankment, although having an Akaike weight of 0.4, has no effect on the response, because the 85% confidence interval of its coefficient included the 0. Environmental variables presented very low Akaike weights, low coefficient values, and many of them also included the 0 within their confidence intervals, therefore, having no effect on the response.

**Table 3:** Averaged model coefficients and variable relative importance estimated from the model selection table (Table 2). Variables are presented within environmental and infrastructure groups

Variable	Models	Σ*w*_i_	Coefficient	85% Confidence interval	
				7.5%	92.5%
Environmental					
axis1	4	0.02	−0.001	−0.218	0.087
axis2	4	0.02	0.002	−0.012	0.188
ndvi	4	0.02	−0.002	−0.249	−0.031
altitude	3	0.02	−0.002	−0.501	0.267
dist.water	3	0.02	0.001	−0.093	0.214
Infrastructure					
adt	4	0.98	0.242	0.045	0.447
adt^2^	4	0.98	−0.576	−0.857	−0.314
cover.herb	4	0.4	0.079	0.042	0.354
embank [Presence]	3	0.4	0.031	−0.106	0.261
verge.width	4	0.99	0.311	0.189	0.437

## 4 DISCUSSION

Our results show that in a disturbed landscape, variables related to human infrastructure—motorways—had a larger explanatory power of rabbit abundance than those related to the environment. Thus, although environmental gradients in the area put the main frame to rabbit populations, the variability in rabbit abundance at a small scale is better explained by the modifications to the habitat caused by motorways. Human disturbance is present in many landscapes (Foley et al., 2005; Sanderson et al., 2002), and it combines with natural conditions to shape wildlife populations. In the case of rabbits, the observed response to the infrastructure could be used as a base for a strategy to manage their populations in verges.

Our analysis highlights the crucial role of road characteristics as important factors for animal response to infrastructures (Farmer & Brooks, 2012; Gomes, Grilo, Silva, & Mira, 2009; Santos et al., 2013). The most relevant infrastructure variables were the width of the verge and traffic volume. Traffic volume had a negative impact on rabbit abundance and increasingly so as the daily number of vehicles grows. The more vehicles, the higher the disturbance, and probability of roadkill when individuals cross the road (Grilo, Ferreira, & Revilla, 2015). Noise from traffic can affect species behavior and increase stress responses, such as a reduction on the time feeding (Shannon, Angeloni, Wittemyer, Fristrup, & Crooks, 2014). Also, roadkill rates increase with traffic flow. For example, in a study in the Iberian Peninsula, a twofold increase in traffic volume increased five times rabbit road mortality (Carvalho & Mira, 2011). Although in small roads, the low density of vehicles can have a positive effect in small mammal density in verges, above a threshold, it becomes negative (Ascensão, LaPoint, & van der Ree, 2015; Gunson, Mountrakis, & Quackenbush, 2011). As our survey focused on motorways, our data already start at medium traffic levels (over 6,000 vehicles per day). The high reproductive rate of rabbits may be an advantage in such situations, as populations can compensate mortality at medium traffic levels while such traffic will have a stronger impact on predators (Benítez‐López et al., 2010; Rytwinski & Fahrig, 2012). This combination of effects may create a predation release effect near motorways (Fahrig & Rytwinski, 2009) that could explain the nonlinear effect of traffic above medium traffic levels denoted by the quadratic relationship. For high traffic volumes, mortality by vehicles will have a larger effect on rabbit populations and the disturbance of the traffic will be higher, with potential negative effects on reproduction due to stress (Navarro‐Castilla et al., 2014).

The other main infrastructure variable outlined by our models is the width of verges, a feature purely dependent on the design of the road and how far from the paved surface the perimeter fence is located. Wider verges provide more habitat for the species and also allow individuals to stay safer, being further away from vehicles and avoiding roadkill (Bellamy, Shore, Ardeshir, Treweek, & Sparks, 2000; de Redon et al., 2015). This is an interesting feature, as it can be easily changed during design or managed after the construction of the infrastructure, at least in those areas that do not include an embankment. A closer look to the data revealed a threshold at 10 m wide verges. Although the 16% of our verges had less than 10 m, only the 6% of transects with relative high rabbit abundance—more than 1,000 pellets—were in those narrow verges, suggesting that dense populations of rabbits generally occur at wider verges.

Another variable supported by our model selection was the vegetation cover in verges. Vegetation in verges may play a key role, as herbaceous plants are an important food source and shrubs proportionate cover against predators (Calvete et al., 2004). We found a positive effect of herbaceous cover, pointing to a higher importance of the availability of a food source than the presence of protection shrubs (they were negatively correlated). In our study, almost all verges presented more than 25% of herbaceous cover so that in areas where vegetation is very scarce, this factor could be a limiting factor for rabbits. Also, this result should be interpreted cautiously, as the high availability of herbs in verges could have overestimated the importance of this variable and the strong correlation with other vegetation types might hide a combined effect of herbs with shrub cover, which is to be expected following other studies (Beja, Pais, & Palma, 2007; Calvete et al., 2004).

Noticeable, a potentially relevant variable of the infrastructure showed no effect in our study, presence of embankments. The presence of embankments could be of importance in other areas where available digging ground for rabbits is limited, as the embankment provides a good substrate for warren building (Gea‐Izquierdo, Munoz‐Igualada, & San Miguel‐Ayanz, 2005), and may benefit rabbit abundance in road verges (Barrientos & Bolonio, 2009). Therefore, it could be a key factor under other circumstances. In addition, embankments are more probable in wider verges and part of the importance of the verge width might be explained by the former.

The environmental variables were not selected as important in our model selection analysis. Environmental variables are those related with the distribution of the species at a broad scale, and thus, variability in rabbit abundance index was better explained by changes in local conditions inside the region. Although with little support, the only environmental variable that had an effect was NDVI, pointing to areas with low productivity or dry vegetation having more rabbits. As NDVI was measured in images taken in summer, it is probably pointing to Mediterranean dry shrubs and pastures, which are the preferred habitat by rabbits (Blanco, 1998).

It was surprising that those areas with natural or seminatural habitat (“dehesas”, with some tree cover) around the motorway did not show a high rabbit abundance in motorway verges. On the contrary, the positive effect of verges on rabbit abundance seems to be related to those areas that are already under human pressure. There, verges can provide a refuge for the species, for example, areas for warren building which are absent in the wider landscape. Thus, it is possible that the relationship of rabbit with verges is more of tolerance rather than preference (Planillo & Malo, 2013). We observed the highest abundances in agricultural areas, where crops provide food for rabbits (Barrio, Bueno, Villafuerte, & Tortosa, 2013), but ploughing destroys their warrens and prevent them to establish in the fieldcrops (Calvete et al., 2004). Similar patterns of species inhabiting verges near agricultural areas have been also observed in mice species (Bellamy et al., 2000; de Redon et al., 2015).

It is important to note that the low variance explained by environmental variables in our analysis reflects the high impact that infrastructure variables have in rabbit abundance. The surveyed motorways captured a wide range of values for studied variables. The environmental variables allowed the presence of rabbits, and thus, their effect in rabbit index variability was not as important as the effect of infrastructure variables that modified local conditions. Combining both types of variables, we obtain that maximum rabbit abundance occurs in areas where the environment allows the survival of rabbit populations and the infrastructure has the optimal characteristics of wide verges and low traffic volume. Another interesting effect is that the infrastructure somewhat has the potential of extending the distribution of the species in human environments, allowing rabbit populations to establish in verges in areas where disturbance in the landscape is excessive for the species survival, as in intensive agricultural areas.

From an applied perspective, our results show that the response of the species is modulated to a high degree by features that can be modified and therefore, used in management to avoid undesirable outcomes (Lees & Bell, 2008). Such preventive action may allow a human growth more compatible with biodiversity protection (McDonald, Kareiva, & Forman, 2008) in a scenario of increasing transportation demand (Dulac, 2013). In addition, as resources are limited, we should prioritize those areas with higher potential for conflict or where prevention or mitigation can be more effective (Farmer & Brooks, 2012; Malo et al., 2004). This will be the case for example of road verges in agricultural areas (this study; Bellamy et al., 2000; de Redon et al., 2015). If desired, the verge management focused to reduce rabbit populations may be counteracted by allowing the species to establish in other points of the landscape, for example, leaving uncultivated plots between field crops (de Redon et al., 2015) or maintaining suitable areas for predators and preys far from roads (Gomes et al., 2009). This may be a real option for rabbits as the species prefer, when available, areas with less disturbance away from motorways (Planillo & Malo, 2013).

The extrapolation of our results to other species should be made with caution. However, as rabbits are widespread and can be found in all continents (Lees & Bell, 2008), our results will have widespread applications in many countries. In addition, the same recommendations could be extended to other species inhabiting road verges, after the examination of each case. In any case, monitoring studies should follow to assess the real outcome and to improve initial measures for any species.

In conclusion, variability in rabbit abundance in motorway verges responds to infrastructure characteristics, or in other words, to local changes in the landscape introduced by human activities. Our results show that controlling the width of the verges may be an effective measure to reduce or boost rabbit populations near motorways, taking into account the traffic flow. For medium traffic volumes, a distance of 10 m from the road surface to perimeter fence seems to be threshold for high density of rabbits. For example, taking into account this distance during road building should prevent problems with high rabbit populations in the future, and for already constructed roads, moving the fence closer to the paved surface should reduce rabbit abundance. In order to preserve the species within its natural range, we suggest to compensate this actions by providing patches of natural habitat away from the road. Also, removing vegetation from verges will discourage the presence of rabbits, as well as other herbivorous species (Bellamy et al., 2000; Sabino‐Marques & Mira, 2011). Besides, other common measures such as building a perimeter fence seemed ineffective for small species, as it was revealed in our comparison between transects inside and outside the fence, including sections that were reinforced with 5 cm wired mesh.

## CONFLICT OF INTEREST

None declared.

## AUTHORS' CONTRIBUTIONS

AP and JEM conceived the ideas and design methodology. AP collected and analyzed the data, and led the writing of the manuscript. All authors contributed critically to the drafts and gave final approval for publication.

## DATA ACCESSIBILITY

Data is available from the Dryad Digital Repository: https://doi.org/10.5061/dryad.c3tg3
