# Supplementary material for: Infrastructure features outperform environmental variables explaining rabbit abundance around motorways
Source: Ecol Evol. 2017 Dec 12;8(2):942–52. doi: 10.1002/ece3.3709 (PMC5773299; doi:10.1002/ece3.3709)
Supplement: Supplementary file 1 [file ECE3-8-942-s001.doc]

**Supporting information**


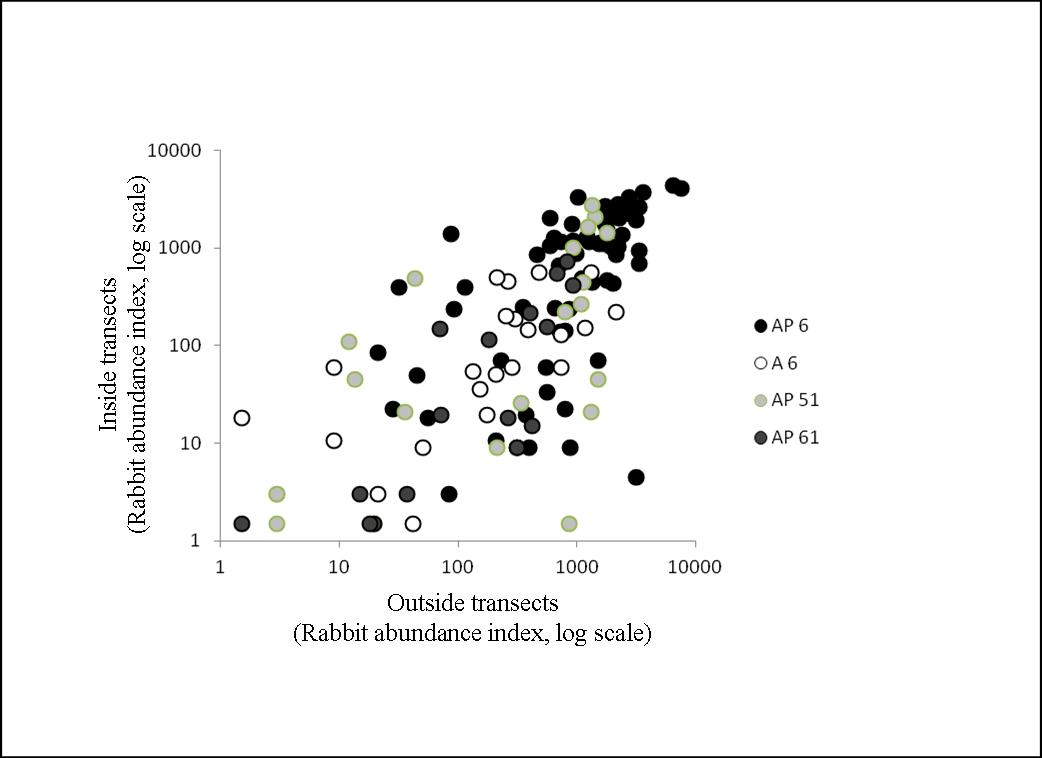


Figure S1. Correlation in rabbit abundance values between inside and outside transects, logaritmic scale.
