# Supplementary material for: Infrastructure features outperform environmental variables explaining rabbit abundance around motorways
Source: Ecol Evol. 2017 Dec 12;8(2):942–52. doi: 10.1002/ece3.3709 (PMC5773299; doi:10.1002/ece3.3709)
Supplement: Supplementary file 2 [file ECE3-8-942-s002.doc]

**Supporting information**


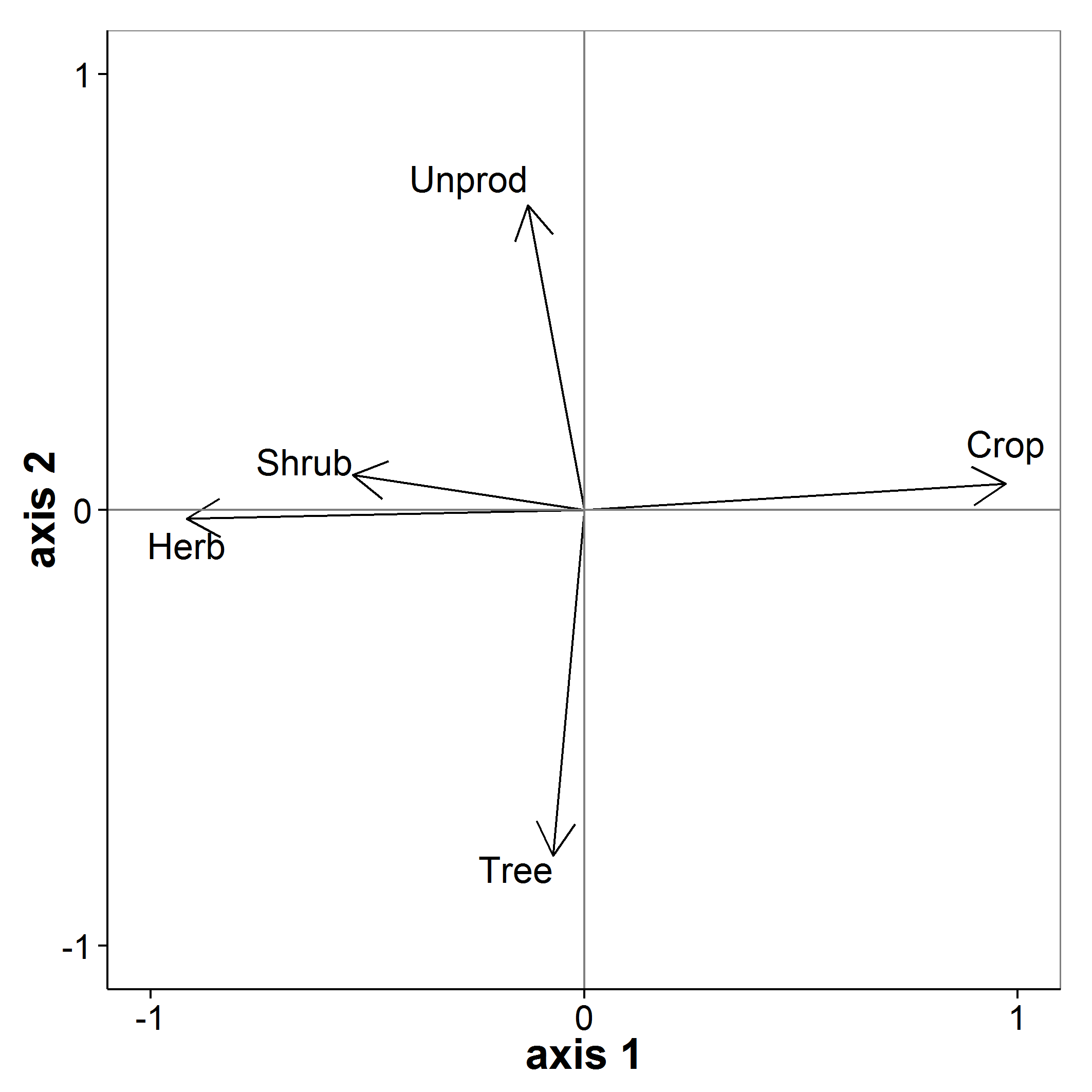


Figure S2. Factor loadings in the PCA of vegetation. Axis 1 explained 42% variance and depicted a gradient from plots with herbaceous (herb) and shrub vegetation to plots dominated by crops. Axis 2 explained 23 % of variance and separated unproductive (unprod) plots from those with tree cover.
