# Supplementary material for: Infrastructure features outperform environmental variables explaining rabbit abundance around motorways
Source: Ecol Evol. 2017 Dec 12;8(2):942–52. doi: 10.1002/ece3.3709 (PMC5773299; doi:10.1002/ece3.3709)
Supplement: Supplementary file 3 [file ECE3-8-942-s003.doc]

**Supporting information**

Table S1. Descriptive data of all variables measured in the study area. Explicative variables split into environment-related variables and infrastructure-related ones. Max: maximum value; Min: minimum value; Avg: average; SD: standard deviation

|  | **RABBIT** | **ENVIRONMENT** | | | | | | | | **INFRASTRUCTURE** | | | | | |
| --- | --- | --- | --- | --- | --- | --- | --- | --- | --- | --- | --- | --- | --- | --- | --- |
|  | **Abundance index** | **Herb cover (%)** | **Shrub cover (%)** | **Crop cover (%)** | **Tree cover (%)** | **Unprod cover (%)** | **NDVI** | **Dist. water (m)** | **Altitude (m)** | **Verge width (m)** | **Herb cover (%)** | **Shrub cover (%)** | **Tree cover (%)** | **Unprod cover (%)** | **adt (vehicles day^-1^)** |
| **Max** | 4662.25 | 100 | 24.28 | 92.06 | 60.09 | 60.66 | 0.02 | 5767.18 | 1312.5 | 79.5 | 100 | 72.5 | 90 | 80 | 31284 |
| **Min** | 0 | 2.46 | 0 | 0 | 0 | 0 | -0.07 | 72.88 | 810.5 | 1 | 7.5 | 0 | 0 | 0 | 6293.64 |
| **Avg.** | 684.59 | 54.48 | 2.69 | 28.42 | 5.13 | 9.29 | -0.03 | 2503.71 | 1069.55 | 18.37 | 68.12 | 13.2 | 10.06 | 8.63 | 18726.8 |
| **SD** | 954.41 | 30.56 | 4.7 | 33.47 | 10.72 | 9.59 | 0.02 | 1566.44 | 142.04 | 11.21 | 29.22 | 18.11 | 20.09 | 15.64 | 10337.8 |
